# Gut microbiota mediated the therapeutic efficacies and the side effects of prednisone in the treatment of MRL/lpr mice

**DOI:** 10.1186/s13075-021-02620-w

**Published:** 2021-09-14

**Authors:** Mingzhu Wang, Zhengyang Zhu, Xiaoying Lin, Haichang Li, Chengping Wen, Jie Bao, Zhixing He

**Affiliations:** 1 Institute of Basic Research in Clinical Medicine, College of Basic Medical Science, Zhejiang Chinese Medical University, Hangzhou, 310053 China; 2 The First School of Clinical Medicine, Zhejiang Chinese Medical University, Hangzhou, 310053 Zhejiang China

**Keywords:** Systemic lupus erythematosus, Gut microbiota, Prednisone, Fecal microbiota transplantation, MRL/lpr mice

## Abstract

**Background:**

Growing evidences indicate that the alterations in gut microbiota are associated with the efficacy of glucocorticoids (GCs) in the treatment of systemic lupus erythematosus (SLE). However, there is no evidence to prove whether gut microbiota directly mediates the effects of GCs.

**Methods:**

Using the MRL/lpr mice, this study firstly addressed the effects of three doses of prednisone on gut microbiota. Then, this study used fecal microbiota transplantation (FMT) to transfer the gut microbiota of prednisone-treated MRL/lpr mice into the blank MRL/lpr mice to reveal whether the gut microbiota regulated by prednisone had similar therapeutic efficiency and side effects as prednisone.

**Results:**

The effects of prednisone on gut microbiota were dose-dependent in the treatment of MRL/lpr mice. After transplantation into MRL/lpr mice, prednisone-regulated gut microbiota could alleviate lupus, which might be due to decreasing *Ruminococcus* and *Alistipes* and retaining the abundance of *Lactobacillus*. However, prednisone-regulated gut microbiota did not exhibit side effects as prednisone. The reason might be that the pathogens upregulated by prednisone could not survive in the MRL/lpr mice as exogenous microbiota, such as *Parasutterella*, *Parabacteroides*, and *Escherichia-Shigella*.

**Conclusions:**

These data demonstrated that the transplantation of gut microbiota may be an effective method to obtain the therapeutic effects of GCs and avoid the side effects of GCs.

**Supplementary Information:**

The online version contains supplementary material available at 10.1186/s13075-021-02620-w.

## Background

Systemic lupus erythematosus (SLE) is an autoimmune disease characterized by the presence of autoantibodies, which cause the formation of immune complexes and inflammation of multiple organs [1]. The clinical manifestations of SLE are varied, including arthritis, renal disease, anemia, rashes, and neuropsychiatric symptoms [2]. Although the pathogenesis of SLE is complex and not clear, genetic, environmental, and hormonal factors contribute to the occurrence of SLE [3]. Standard clinical therapies for SLE are glucocorticoids combined with immunosuppressive agents, antimalarial drugs, and non-steroidal anti-inflammatory drugs [4]. At the same time, growing evidence demonstrates that gut microbiota plays a significant role in SLE development and treatment [5].

Glucocorticoids (GCs) are effective and commonly used anti-inflammatory and immunosuppressive agents. In addition to SLE, GCs are used to manage inflammatory diseases including inflammatory bowel disease, rheumatoid arthritis, and chronic renal diseases as well as some cancers [6, 7]. While the side effects seen in many organ systems limit GCs’ full dose range potential and long-term use. GC-associated side effects may be musculoskeletal, endocrine, gastrointestinal, neuropsychiatric, cardiovascular, dermatologic, ocular, or immunologic [8]. Recent studies have shown that some part of the signaling pathways related to GCs is due to their effects on gut microbiota [9, 10]. Thus, GCs may play therapeutic roles by altering the gut microbiota.

Gut microbiota inhabits the intestine, forming a complex ecological community that influences normal physiology and susceptibility to disease through its collective metabolic activities and host interactions [11]. In the treatment of SLE by GCs, gut microbiota not only affects its efficiency but also serves as a therapeutic target [12, 13]. However, little is known about whether gut microbiota plays role in the side effects of GCs in SLE.

Firstly, to clarify how GCs regulates the gut microbiota, female MRL/lpr mice were administered for a long duration with three doses of prednisone. Then, the feces of prednisone-treated MRL/lpr mice were transplanted into the MRL/lpr mice to reveal the effects of prednisone-altered gut microbiota on mice. Illumina Miseq sequencing was used to explore the alterations in gut microbiota caused by GCs.

## Methods

### Animals and chemicals

This study used the MRL/MpJ-Fas lpr (MRL-lpr) mouse strain as the model mouse for SLE. Female MRL/lpr mice were purchased from Shanghai SLAC Laboratory Animal Co., Ltd., at 7 weeks of age. All the mice were allowed to be acclimated to our animal facility for 1 week and then randomly divided into different groups in the specific-pathogen-free environment of Zhejiang Chinese Medical University laboratory animal research center. Mice were housed under a 12-h/12-h light/dark cycle and constant temperature (25 ± 1^o^C) and humidity (50 ± 5%) with food and water available ad libitum. All animal experiments were performed according to the requirements of the Institutional Animal Care and Use Committee of China.

The prednisone (purity ≥ 99.0%, Sigma-Aldrich, United States) was administered from 8 to 16 weeks of age. Mice were weighed twice weekly, and the drug doses were adjusted accordingly.

### Experimental design to reveal the effects of prednisone on gut microbiota in MRL/lpr mice

The MRL/lpr mice were grouped into four groups (seven per group): (1) low-dose prednisone (Pred-2.5): oral gavage with 2.5 mg prednisone/kg of the body per day; (2) middle dose prednisone (Pred-5): oral gavage with 5 mg prednisone/kg of the body per day); (3) high-dose prednisone (Pred-10): oral gavage with 10 mg prednisone/kg of the body per day; and (4) model group (MT): oral gavage with sterile water per day. The time course and grouping information was shown in Figure S1. The entire experimental period was 8 weeks. In addition, the 8 weeks-old C57BL/6 mice were given oral gavage with sterile water per day as the control group (CT) throughout the experiment.

The blood was obtained from the eye socket vein at 12 h after the last drug administration and then centrifuged at 3000 rpm for 15 min at 4^o^C for serum. After the blood collection was completed, the MRL/lpr mice with 16 weeks old were euthanized to obtain the fecal material, spleen, liver, and tibial bone tissues. Fecal material was removed from the colon and stored at −80^o^C for further analysis. The spleen tissue was immediately weighed and ground to prepare cell suspensions. The liver tissue was immediately snap-frozen in liquid nitrogen and stored at −80^o^C. The tibial bone tissue was immediately stripped of the above musculature and stored at 4^o^C.

### Experimental design to reveal the effects of prednisone-altered gut microbiota on MRL/lpr mice

After clarifying the effects of prednisone on gut microbiota, this study employed fecal microbiota transplantation (FMT) to reveal the effects of the prednisone-altered gut microbiota on MRL/lpr mice. The female MRL/lpr mice (8 weeks old) were grouped into three groups (seven per group): MT (oral gavage with sterile water per day), Pred (oral gavage with 10 mg prednisone/kg of the body per day), and FMT (oral gavage with a 200 μL/day aliquot of fecal suspensions). FMT started from the second week of oral gavage prednisone. The time course and grouping information was shown in Figure S2. The entire experimental period was 8 weeks.

The fresh feces were daily collected from the prednisone treated MRL/lpr mice before the administration of prednisone, then were resuspended in 5 times (weight/volume) phosphate-buffered saline solution. The fecal suspensions were passed through a 20-mm filter to remove large particulate, and then, the filtrates were transferred into the mice of the FMT group on the same day. The experiment period and sampling methods were the same as above.

### Lupus activity evaluation

Serum anti-nuclear antibodies (ANA) and anti-double-stranded DNA (dsDNA) were measured by ELISA method using mouse anti-dsDNA antibody (IgG) ELISA Kit (CUSABIO, Wuhan, China) and mouse anti-nuclear antibody (IgG) ELISA Kit (CUSABIO, Wuhan, China) that was based on double antigen sandwich ELISA method. Serum blood urea nitrogen (BUN) was measured based on an enzymatic-colorimetric method by using standard test kits on TBA-40FR automatic biochemical analyzer (Toshiba Medical Sys-terms Co., Ltd., Tokyo, Japan).

Spleen tissues of the mouse were collected to prepare the spleen cell suspension. Cell suspensions were prepared from the spleens and blocked in the presence of anti-CD16/CD32 at 4°C for 15 min, and GC B cells were stained with FITC-anti-CD19, PE-anti-CD3, PE-Cy5-B220, and GL-7 biotin with PE/Cy7 streptavidin. Plasma cells were incubated with FITC-anti-CD3, PE-anti-CD138, and PE-Cy5-B220. DN T cells were stained with anti-CD3, anti-CD4, and anti-CD8 antibodies. For the staining of surface antigens, cells were incubated with antibodies 30min on ice and washed and analyzed by CytoFLEX S Flow cytometer (Beckman Coulter, Inc.) All the results were analyzed using Flowjo software. The antibodies mentioned above were applied according to the manufacturer’s instructions. All antibodies and reagents were purchased from BD Biosciences or BioLegend.

The kidney tissue was also harvested from exsanguinated mice, flushed with 1 x PBS, dissected longitudinally, and fixed in 4.0% formaldehyde overnight, and decalcified in EDTA decalcification solution. The tissues were then embedded in paraffin. Sections of 5 μm were cut from paraffin-embedded tissues and stained with hematoxylin and eosin (H&E) to evaluate the damage of kidney tissue.

### Prednisone’s adverse effects evaluation

This study employed eight indices to reflect the adverse effects of prednisone on the MRL/lpr mice including liver superoxide dismutase (T-SOD), CuZn superoxide dismutase (CuZn-SOD), malonaldehyde (MDA), interleukin-6 (IL-6), and tumor necrosis factor (TNF-α) concentrations, serum fasting blood glucose (FBG), and cholesterol (CHOL) concentrations and bone mineral density (BMD).

The liver tissue was minced, homogenized in ice-cold physiological saline by a glass homogenizer, and centrifuged at 2000 rpm for 15 min at 4°C to afford the 10% (w/v) liver homogenate. The levels of T-SOD, CuZn-SOD, MDA, and total proteins in the liver homogenate were determined using the commercia kits (Nanjing Jiancheng Biotechnic Institute, Nanjing, China). The levels of IL-6 and TNF-α in the liver homogenate were determined using the commercial kits (Multisciences, Hangzhou, China). Serum FBG and CHOL were measured based on an enzymatic-colorimetric method by using standard test kits on TBA-40FR automatic biochemical analyzer (Toshiba Medical Sys-terms Co., Ltd., Tokyo, Japan). BMD was assessed using the Faxitron X-ray with 5.0 kV for 6.0 s.

### Gut microbiota analysis

Stool samples were collected within 10 min after euthanization. Total genomic DNA was extracted from each stool sample using the Fecal DNA Extraction Kit (BioTeke Corporation, Beijing, China) according to the manufacturer’s protocols. DNA extracts were determined by agarose gel electrophoresis (1% w/v agarose) and quantified using a nanodrop 2000 spectrophotometer (Thermo Fisher Scientific). Qualified DNA was PCR amplified with broad-range bacterial primers targeting the V3–V4 regions of the 16S rRNA gene as previously described [14]. Subsequently, the amplicons were purified according to standard procedures, quantified, pooled, and sequenced with the Miseq Reagents Kit v3 (600 cycles, Illumina) according to the manufacturer’s instructions. The sequencing reaction was conducted by Hangzhou Legenomics Bio-Pharm Technology Co., Ltd., Zhejiang, China.

After sequencing, generated FASTQ data of the MRL/lpr mice were prepared for analysis using Quantitative Insights Into Microbial Ecology (QIIME, Version 1.9) [15]. The clean reads were extracted from the raw-paired end reads according to previous studies [14]. UCLUST was used to cluster sequencing reads into operational taxonomical units with a 97% similarity cutoff [16]. Bacterial taxonomy was assigned by using the SILVA [17] and NCBI databases [18]. The OTUs with less than 0.05% sequences of the total number of reads, or present in one sample, were filtered out. The microbiota OTUs table was imported into R software, and the alpha and beta diversity metrics were computed using the “vegan” package. To analyze the alpha diversity, Shannon and Chao1 indices were performed by using R software. For the beta diversity analysis, the principal coordinate analysis (PCoA) based on the unweighted UniFrac distance matrices were visualized by R software. To reveal differences in deeper data of microbial diversity between the samples, a significant test was conducted with the linear discriminant analysis (LDA) effect size (LEfSe) method [19], with a set logarithmic LDA score of 2.0. Additionally, the metabolic function of gut microbiota was inferred using the PICRUST that predicted the molecular functions of each sample based on 16S rRNA marker gene sequences [20]. These predictions were pre-calculated for genes in the KEGG database. To reveal the different predictive functions, Welch’s *t* tests were used for two-group comparisons in STAMP software [21]. The significantly different functions between the two groups were obtained after filtering with a *p* value <0.05.

## Results

### Therapeutic efficiency and side effects of prednisone in the treatment of MRL/lpr mice

The results of the therapeutic efficiency of prednisone were shown in Figure S3. Serum anti-dsDNA and the percentages of plasma, GC B, and DN T cells in the spleen were significantly reduced by all three doses of prednisone after 8 weeks of treatment (Figure S3). Although all three doses of prednisone reduced the serum ANA level, only the high dose of prednisone showed a significant therapeutic efficiency (Figure S3).

**Fig. 1:**
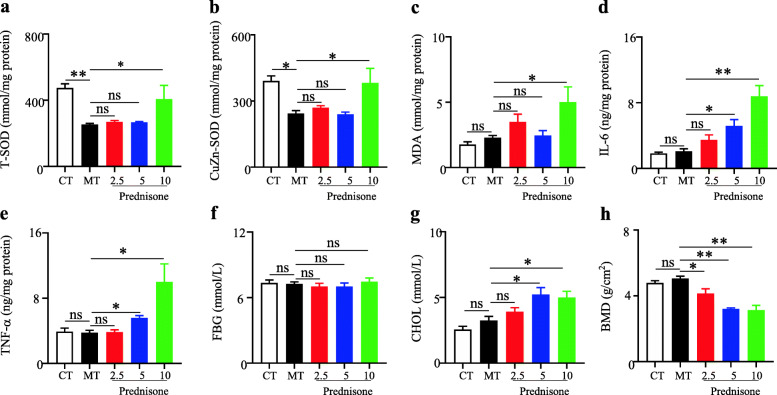
Effects of prednisone on liver total superoxide dismutase (**a**), CuZn superoxide dismutase (**b**), malonaldehyde (**c**), IL-6 (**d**) and TNF-α (**e**), serum fasting blood-glucose (**f**) and cholesterol (**g**), and tibial bone mineral density (**h**) in the treatment of MRL/lpr mice. CT, the C57BL/6 mice treated with sterile water; MT, the MRL/lpr mice treated with sterile water. “**” represents *p* < 0.01; “*” represents *p* < 0.05; “ns” represents no significant difference

To reveal whether the side effects of prednisone were associated with gut microbiota, this study evaluated the effects of prednisone on the liver antioxidant system and inflammation, BMD, serum FBG, and CHOL in MRL/lpr mice. As shown in Fig. 1, the liver CuZn-SOD and T-SOD levels were significantly decreased in MRL/lpr mice compared with the C57BL/6 mice. As one of the antioxidant enzymes, SOD could scavenge oxygen free radicals to protect cells from damage. SOD was excessively consumed by scavenging free radicals, resulting in a corresponding decrease in SOD content, but the high-dose prednisone could significantly induce the increases of T-SOD and CuZn-SOD in MRL/lpr mice, as one type of feedback regulation response. Besides, the antioxidant system could also be reflected by MDA level. As an oxidation product, the significant increase in liver MDA indicated the side effects caused by high-dose prednisone. Two liver inflammation cytokines (IL-6 and TNF-α) and serum CHOL levels showed significant increases in middle and high doses of prednisone-treated mice in comparison with the blank model MRL/lpr mice. In addition, all three doses of prednisone could significantly reduce BMD but had no effects on serum FBG in the treatment of MRL/lpr mice.

In summary, the therapeutic efficiency and side effects of prednisone were positively associated with the dose in MRL/lpr mice.

### Prednisone induced the alterations in gut microbiota

**Fig. 2:**
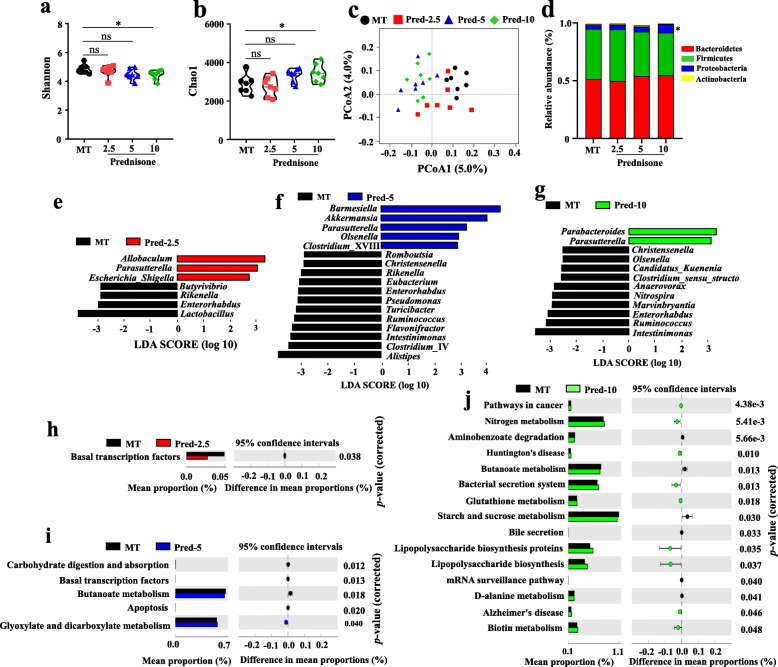
Alterations in gut microbiota caused by prednisone in MRL/lpr mice. Alpha diversity indexes [Shannon (**a**); Simpson (**b**)], PCoA score plots (**c**), and identified major phylum (**d**) in MRL/lpr mice. LEfSe and PICRUST analysis identified the significantly altered genus and KEGG pathways caused by low (**e**, **h**), middle (**f**, **i**), and high doses (**g**, **j**) of prednisone in the treatment of MRL/lpr mice

To clarify whether the alleviation of lupus by prednisone was associated with gut microbiota, the bacterial 16S rRNA v3–v4 regions in colon feces were sequenced. The alpha diversity indices for the Shannon and Chao1 were only significantly different in MT vs. Pred-10 (Fig. 2a, b). A scatter plot based on PCoA scores showed a clear separation of the community composition in MT vs. Pred-5 and MT vs. Pred-10 (Fig. 2c). There was an overlap between the samples of MT and Pred-2.5 (Fig. 2c). At the phylum level, Bacteroidetes appeared to be the most abundant in all four groups, followed by Firmicutes, Proteobacteria, and Actinobacteria (Fig. 2d). One-way ANOVA analysis demonstrated that the only significant difference was the abundance of Proteobacteria between MT and Pred-10.

As compared with the MT group, 7, 17, and 12 markedly different genera were observed in Pred-2.5, Pred-5, and Pred-10 groups, respectively (Fig. 2e–g). Genus *Parasutterella* significantly increased and genus *Enterorhabdus* significantly decreased in all three prednisone treatment groups in comparison with the MT group. In addition, decreased *Rikenella* were shared by Pred-2.5 and Pred-5 groups; decreased *Christensenella*, *Ruminococcus*, and *Intestinimonas* were shared by Pred-5and Pred-10 groups.

Alterations in bacterial taxa also caused the fluctuation of potential metabolic functions of gut microbiota. As compared with the MT group, 1, 5, and 15 markedly different metabolic functions were observed in Pred-2.5, Pred-5, and Pred-10 groups, respectively (Fig. 2h–j). The 2.5 mg/kg Prednisone only caused the decrease of the basal transcription factors pathway. The 5 mg/kg prednisone caused the downregulation of four pathways (basal transcription factors pathway, butanoate metabolism pathway, apoptosis pathway, and carbohydrate digestion and absorption pathway) and the upregulation of glyoxylate and dicarboxylate metabolism pathway. In addition, disease pathways (pathways in cancer, Huntington’s disease, Alzheimer’s disease) and stress-related pathways (bacterial secretion system, glutathione metabolism, biotin metabolism, lipopolysaccharide biosynthesis proteins, and lipopolysaccharide biosynthesis) were upregulated by 10 mg/kg prednisone. In addition, the downregulated pathways caused by 10 mg/kg prednisone were mainly basal metabolism pathways, including butanoate metabolism pathway, starch, and sucrose metabolism pathway, bile secretion pathway, mRNA surveillance pathway, and d-alanine metabolism pathway.

Collectively, the above results indicated that the alterations of gut microbiota were positively associated with the dose of prednisone in the treatment of MRL/lpr mice.

### FMT caused the alterations in gut microbiota

**Fig. 3:**
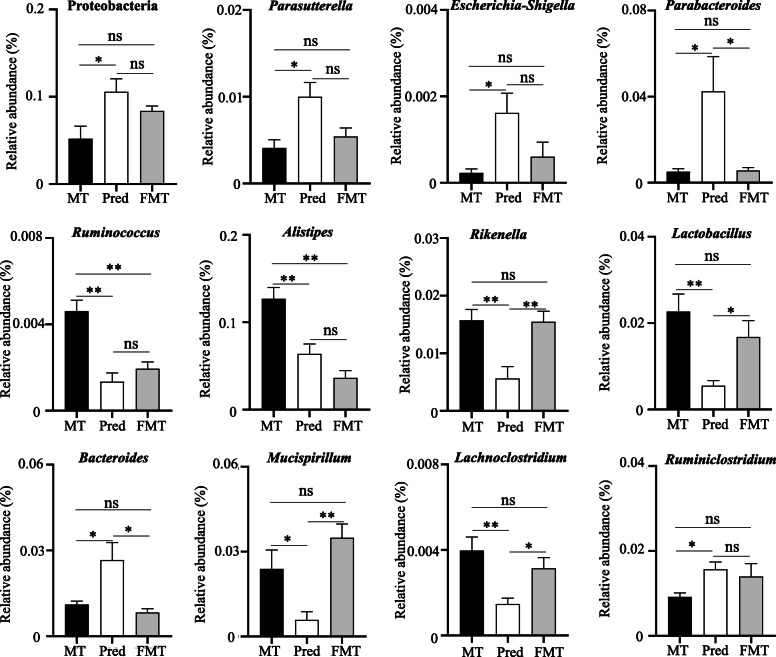
Significantly different genera among MT, Pred, and FMT groups. MT, the MRL/lpr mice treated with sterile water; Pred, the MRL/lpr mice treated with prednisone; FMT, the MRL/lpr mice treated with fecal microbiota transplantation. “**” represents *p* < 0.01; “*” represents *p* < 0.05; “ns” represents no significant difference

As shown in Figure S4a, a scatter plot based on PCoA analysis showed a clear separation among the samples of three groups, indicating the differences in gut microbiota. Consistent with the results shown in Figs. 2 and 3 also demonstrated that prednisone could increase the abundances of phylum Proteobacteria and genus *Parasutterella*, *Escherichia-Shigella*, and *Parabacteroides*, decrease the abundances of *Ruminococcus*, *Alistipes*, *Rikenella*, and *Lactobacillus* in the treatment of MRL/lpr mice. The comparison between MT and FMT indicated that FMT could not transfer the effects of prednisone on phylum Proteobacteria and genus *Parasutterella*, *Escherichia-Shigella*, *Parabacteroides*, *Rikenella*, *Lactobacillus*, *Bacteroides*, *Mucispirillum*, *Lachnoclostridium*, and *Ruminiclostridium* into MRL/lpr mice, but successfully retain the effects of prednisone on genus *Ruminococcus* and *Alistipes* (Fig. 3, Figure S4). Even so, there were also some differences between Pred and FMT groups. Compared with prednisone-treated mice, FMT-treated mice had significantly decreased genus *Parabacteroides* and *Bacteroides* and increasing genus *Rikenella*, *Lactobacillus*, *Mucispirillum*, and *Lachnoclostridium* (Fig. 3, Figure S4).

### Effects of altered gut microbiota by prednisone on lupus activity

**Fig. 4:**
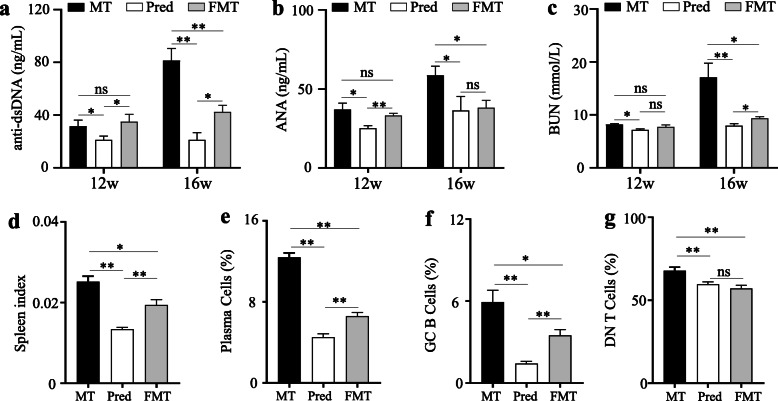
The differences in the lupus activity of MRL/lpr mice among MT, Pred, and FMT groups. **a** Serum anti-dsDNA autoantibody levels. **b** Serum ANA autoantibody levels. **b** Serum urea nitrogen (BUN) levels. **d** Spleen index. **e** Percentage of plasma cells in the spleen. **f** Percentage of GC B cells in the spleen. **g** The percentage of double negative (DN) T cells in the spleen. MT, the MRL/lpr mice treated with sterile water; Pred, the MRL/lpr mice treated with prednisone; FMT, the MRL/lpr mice treated with fecal microbiota transplantation. “**” represents *p* < 0.01; “*” represents *p* < 0.05; “ns” represents no significant difference

As shown in Fig. 4, prednisone could significantly alleviate lupus activity at 12 weeks and 16 weeks in MRL/lpr mice. This study also revealed the effects of altered gut microbiota by prednisone on lupus activity. Compared to the model mice, FMT-treated mice exhibited no difference in serum anti-dsDNA, ANA, and BUN levels at 12 weeks old, but a significant decrease at 16 weeks old (Fig. 4a–c), in spite of the curative effects were not as good as the prednisone-treated mice. Spleen index and its immune cell percentage also indicated that FMT could slightly alleviate lupus through transplanting the gut microbiota of prednisone-treated mice at 16 weeks old (Fig. 4d–g, Figure S5). Additionally, Figure S6 indicated that the transplantation of prednisone-regulated gut microbiota could alleviate skin lesions and kidney inflammatory cell infiltration in MRL/lpr mice. In sum, the altered gut microbiota by prednisone could be beneficial for alleviating lupus, but its effect was weaker than that of prednisone.

### Adverse effects of altered gut microbiota by prednisone in MR/lpr mice

**Fig. 5:**
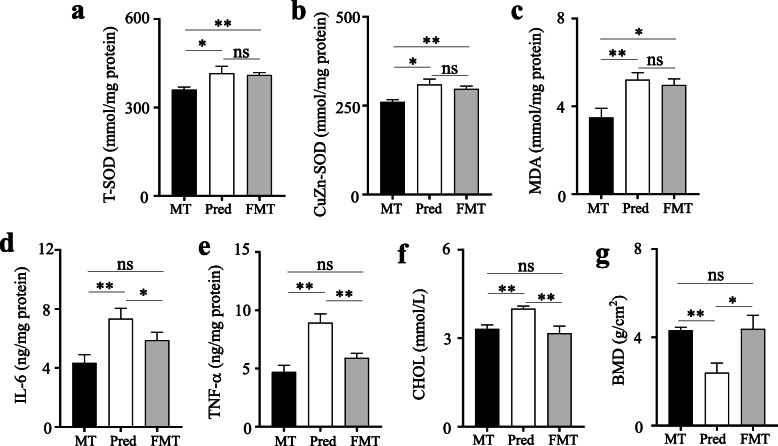
The differences in the side effects of prednisone among MT, Pred, and FMT groups. **a** Liver total superoxide dismutase (T-SOD) activity. **b** Liver CuZn superoxide dismutase (CuZn-SOD) activity. **c** Liver malonaldehyde (MDA) concentration. **d** Liver IL-6 concentration. **e** Liver TNF-α concentration. **f** Serum cholesterol (CHOL) concentration. **g** Tibial bone mineral density (BMD). MT, the MRL/lpr mice treated with sterile water; Pred, the MRL/lpr mice treated with prednisone; FMT, the MRL/lpr mice treated with fecal microbiota transplantation. “**” represents *p* < 0.01; “*” represents *p* < 0.05; “ns” represents no significant difference

Compared to the model MRL/lpr mice, both prednisone-treated and FMT-treated mice exhibited significant increases in T-SOD, CuZn-SOD activity, and MDA level, indicating the elevating antioxidant activity after two different treatments (Fig. 5a–c). The increased antioxidant activity of FMT-treated mice might be induced by exogenous gut microbiota. Additionally, prednisone could cause a significant increase in liver inflammatory cytokines (IL-6, TNF-α), cholesterol levels, and bone density in MRL/lpr mice, but the altered gut microbiota by prednisone has no the above effects (Fig. 5d–g). In sum, the altered gut microbiota by prednisone did not exhibit the side effects of prednisone in MRL/lpr mice.

## Discussion

The gut microbiota affects not only the activity of lupus but also the treatment effects of lupus with glucocorticoids [12, 22]. At the same time, one of the mechanisms of GCs is regulating the gut microbiota to play the therapeutic effect [23, 24]. It is well known that GCs also cause side effects while alleviating the conditions of disease. The previous studies were limited to revealing the correlation between gut microbiota and the efficacy or side effects of GCs [9, 13]. To our knowledge, there was no study demonstrating whether gut microbiota directly mediates the therapeutic efficiency and side effects of GCs. This study was the first to reveal whether the gut microbiota altered by GCs played a similar role as GCs in the treatment of MRL/lpr mice.

This study also revealed the disturbing effects of GCs on the gut microbiota, and for the first time showed that these disturbance effects were positively correlated with GC concentration. At the phylum level, prednisone could significantly upregulate the abundance of Proteobacteria in the treatment of MRL/lpr mice. A recent study has also reported that dexamethasone treatment caused an increased abundance of Proteobacteria [10]. Phylum Proteobacteria was closely related to a “pro-inflammatory” state of the host, transferred the body to a susceptible status [25, 26]. At the genus level, genus *Parasutterella* significantly increased in all three prednisone-treated MRL/lpr mice, which was associated with chronic intestinal inflammation in irritable bowel syndrome [27]. Prednisone also caused the increases of some pathogens in the treatment of MRL/lpr mice, such as *Parabacteroides* [28] and *Escherichia-Shigella* [29]. In addition, the PICRUST analysis indicated that prednisone enhanced the disease-associated function and downregulated basal metabolism function of gut microbiota. Therefore, the altered gut microbiota by prednisone was associated with prednisone’s side effects.

Additionally, the regulated microbiota by prednisone also mediated the therapeutic efficiency of prednisone in the treatment of MLR/lpr mice. Genus *Ruminococcus* could produce a B cell superantigen postulated to contribute to immune pathogenesis of SLE [30]. Genus *Alistipes* was over-represented in SLE patients [30], which could regulate T cell differentiation [31]. Genus *Rikenella* was positively correlated with lupus activity in lupus mice [14] and patients [32]. Moreover, the alterations in gut microbiota caused by prednisone might regulate the formation of GC B cells and the differentiation of plasma cells to alleviate lupus [33]. Therefore, prednisone could exert its curative effect through regulating gut microbiota in MRL/lpr mice.

The above conclusions well demonstrated that the gut microbiota mediated the therapeutic efficiency and sides effects of prednisone in the treatment of MRL/lpr mice. Next, this study transplanted prednisone-regulated gut microbiota into blank MRL/lpr mice and found that the gut microbiota could play a role in the remission of lupus but do not cause the sides effects as prednisone. The residual concentration of prednisone in feces indicated that the above effects of the prednisone-regulated gut microbiota were not due to residual prednisone in feces (Figure S7). Further analysis of the altered gut microbiota by FMT might explain the effect of prednisone-regulated gut microbiota in MRL/lpr mice. FMT could cause the decrease of *Ruminococcus* and *Alistipes* and the increase of *Lactobacillus.* Genus *Lactobacillus* was beneficial for alleviating lupus but downregulated by prednisone [34, 35]. Besides, *Lactobacillus* might inhibit the proliferation of B cells (including GC B and plasma cells) and T cells to alleviate lupus in MRL/lpr mice [36]. However, FMT did not cause the increases of some pathogens induced by prednisone in MRL/lpr mice. The reason might be that the endogenous gut microbiota in MRL/lpr mice could resist the invasion of exogenous pathogens in the prednisone-regulated gut microbiota.

## Conclusions

In conclusion, this study successfully indicated that gut microbiota directly mediated the therapeutic efficiency and side effects of GCs, and further clarified that the transplantation of gut microbiota may be an effective method to obtain the therapeutic effects of GCs and avoid the side effects of GCs. However, this study did not validate the results through human samples and further reveal the relevant mechanism from the perspective of the host. Even so, this study is the first to reveal gut microbiota directly mediating the therapeutic efficiency of GCs.

## Supplementary Information

**Additional file 1.**

## Data Availability

The raw sequences of 16S v3–v4 regions from MRL/lpr mice have been submitted to the NCBI Sequence Read Archive under accession numbers SRR14038709-SRR14038736 and SRR14038813-SRR14038832.

## Abbreviations

SLE: Systemic lupus erythematosus
GCs: Glucocorticoids
FMT: Fecal microbiota transplantation
MRL-lpr: MRL/MpJ-Fas lpr
MT: The MRL/lpr mice treated with sterile water
Pred-2.5: The MRL/lpr mice treated with 2.5 mg prednisone/kg of the body per day
Pred-5: The MRL/lpr mice treated with 5.0 mg prednisone/kg of the body per day
Pred-10: The MRL/lpr mice treated with 10 mg prednisone/kg of the body per day
Pred: The MRL/lpr mice treated with prednisone
FMT: The MRL/lpr mice treated with fecal microbiota transplantation
ANA: Anti-nuclear antibodies
anti-dsDNA: Anti-double-stranded DNA
T-SOD: Total superoxide dismutase
CuZn-SOD: CuZn superoxide dismutase
MDA: Malonaldehyde
IL-6: Interleukin-6
TNF-α: Tumor necrosis factor
FBG: Fasting blood-glucose
CHOL: Cholesterol
BMD: Bone mineral density
QIIME: Quantitative Insights Into Microbial Ecology
NCBI: National Center for Biotechnology Information
OTUs: Operational taxonomic units
PCoA: Principal coordinate analysis
LDA: Linear discriminant analysis
LEfSe: Linear discriminant analysis effect size
PICRUST: Phylogenetic investigation of communities by reconstruction of unobserved states
KEGG: Kyoto Encyclopedia of Genes and Genomes
GC B cells: Germinal center B cells
DN T cells: Double-negative T cells
