# Supplementary material for: Gut microbiota mediated the therapeutic efficacies and the side effects of prednisone in the treatment of MRL/lpr mice
Source: Arthritis Res Ther. 2021 Sep 14;23:240. doi: 10.1186/s13075-021-02620-w (PMC8439074; doi:10.1186/s13075-021-02620-w)
Supplement: Supplementary file 1 — Additional file 1. [file 13075_2021_2620_MOESM1_ESM.pdf]

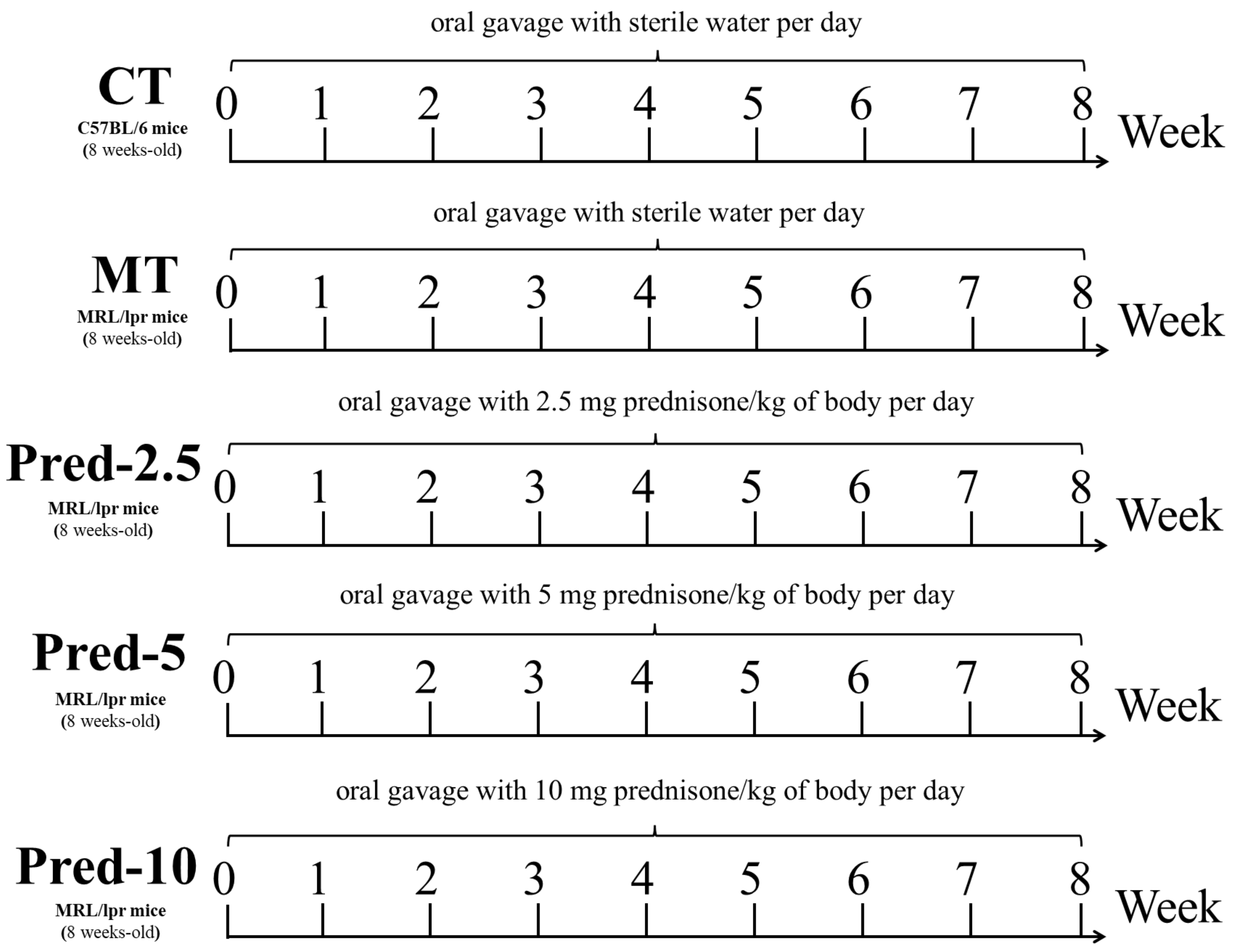

Figure S1 The diagram of the experiment treatments to reveal the effects of prednisone on gut microbiota in MRL/lpr mice. CT, the C57BL/6 mice treated with sterile water; MT, the MRL/lpr mice treated with sterile water.

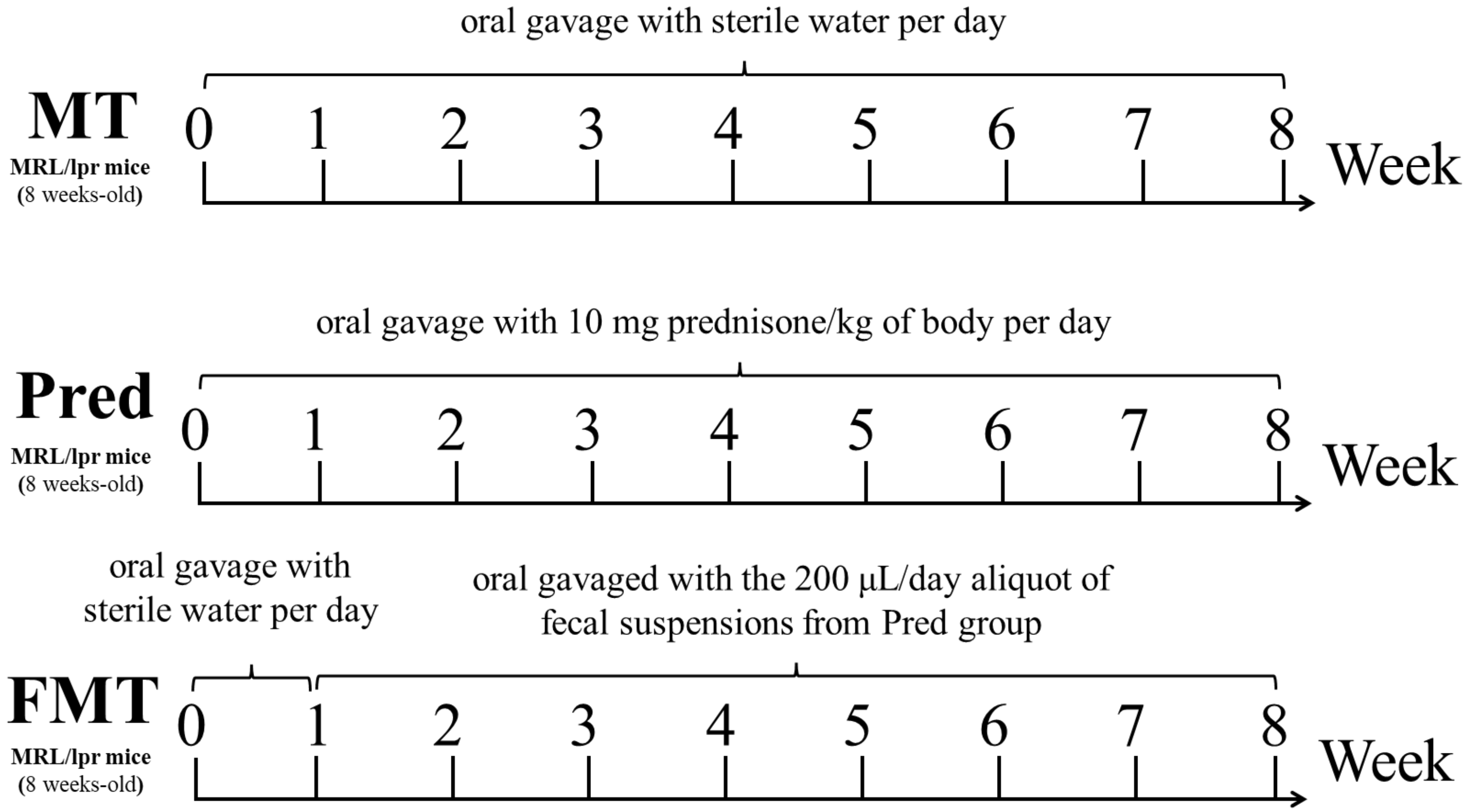

Figure S2 The diagram of the experiment treatments to reveal the effects of prednisone-altered gut microbiota on MRL/lpr mice. MT, the MRL/lpr mice treated with sterile water; Pred, the MRL/lpr mice treated with prednisone; FMT, the MRL/lpr mice treated with fecal microbiota transplantation.

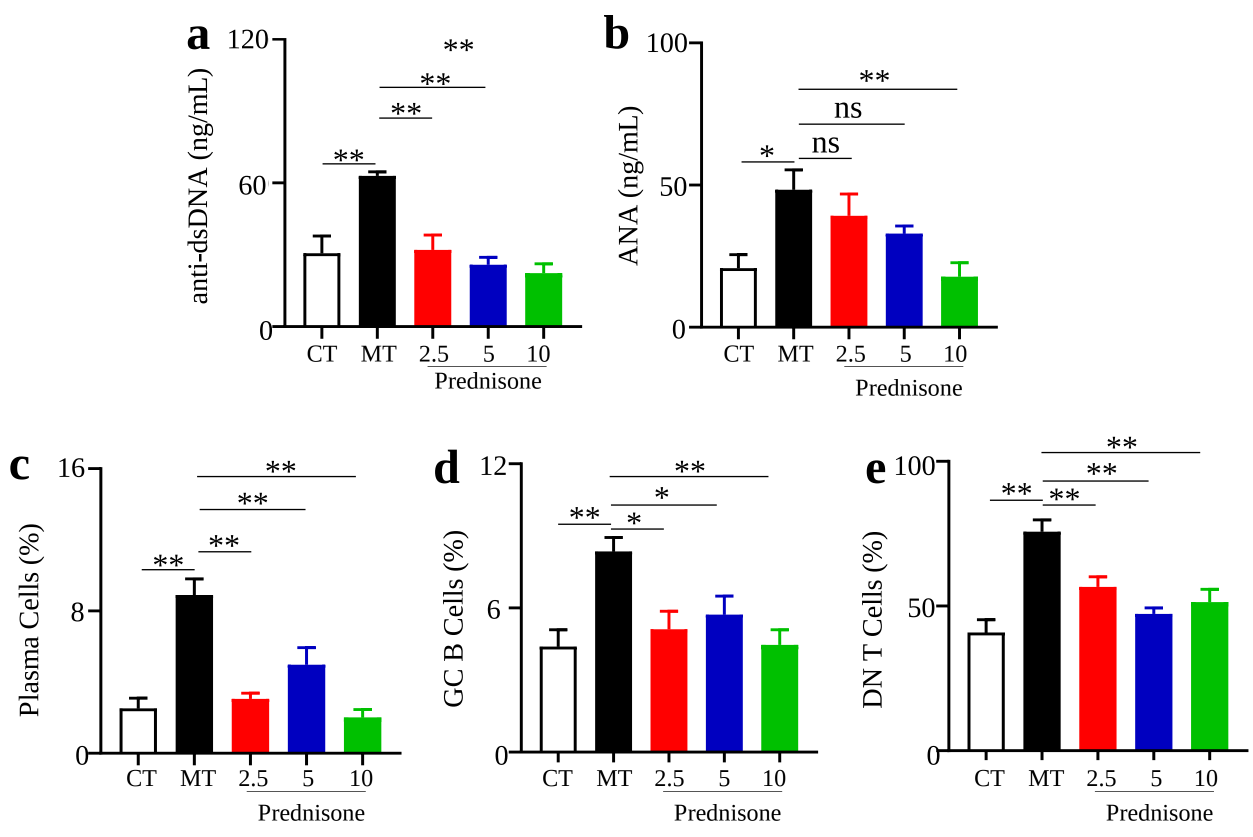

Figure S3 Effects of prednisone on lupus activity. (a) Serum anti-dsDNA concentration. (b) Serum ANA concentration. (c) The percentage of plasma cells in spleen. (d) The percentage of germinal center (GC) B cells in spleen. (e) The percentage of double negative (DN) T cells in spleen. "**" represents $p<0.01$; "*" represents $p<0.05$; "ns" represents no significant difference.

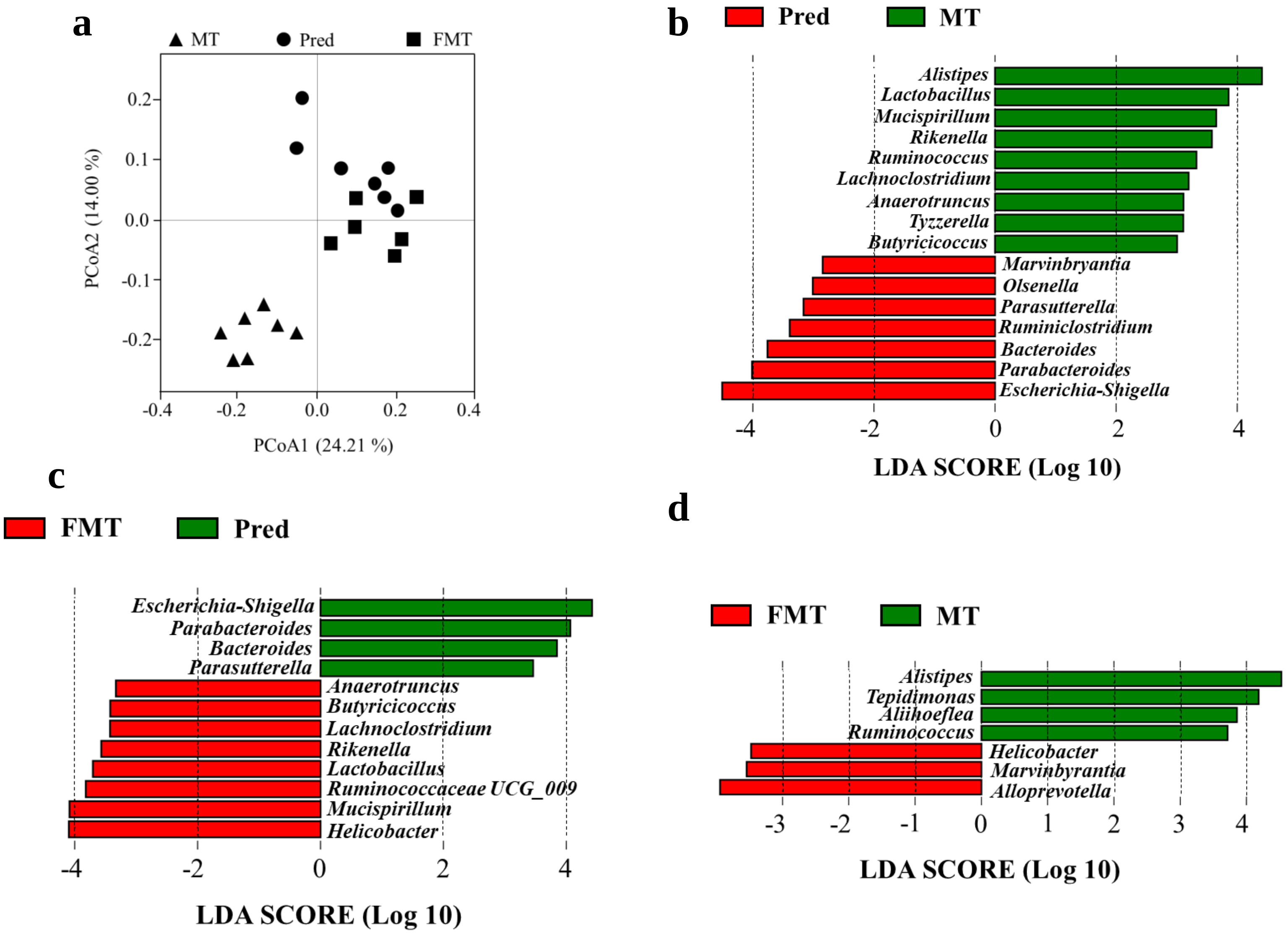

Figure S4 (a) PCoA score plots based on gut microbiota compositions in MRL/lpr mice; LEfSe analysis identified the significantly altered genus in MT *vs.* Pred (b), Pred *vs.* FMT (c), and MT *vs.* FMT (d). MT, the MRL/lpr mice treated with sterile water; Pred, the MRL/lpr mice treated with prednisone; FMT, the MRL/lpr mice treated with fecal microbiota transplantation.

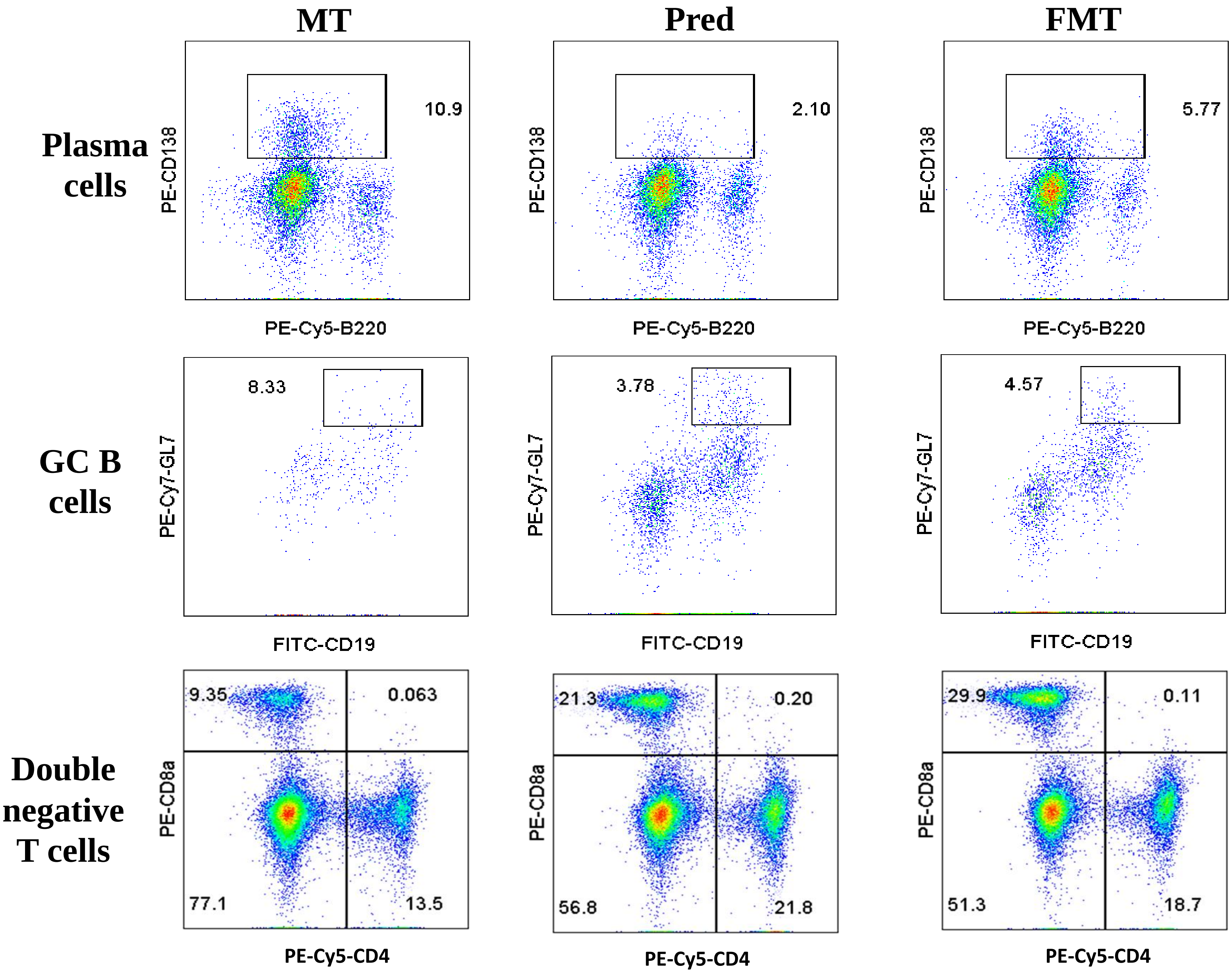

Figure S5 Representative images of flow cytometry analysis for plasma, GC B and double negative T cells in MRL/lpr mice. MT, the MRL/lpr mice treated with sterile water; Pred, the MRL/lpr mice treated with prednisone; FMT, the MRL/lpr mice treated with fecal microbiota transplantation.

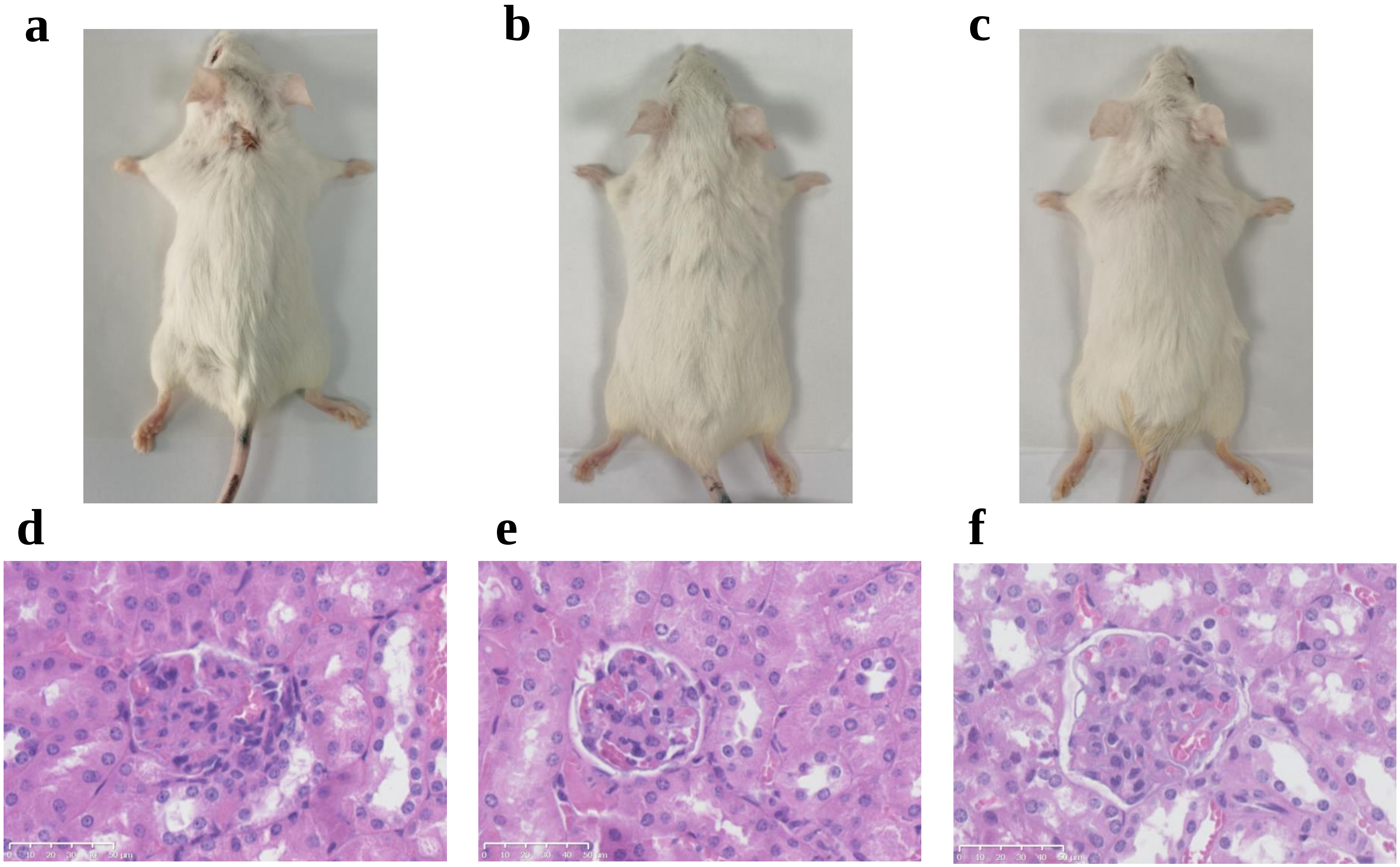

Figure S6 Representative images of skin lesions (a: MT; b: Pred; c: FMT) and H&E staining from kidneys (d: MT; e: Pred; f: FMT)) in MRL/lpr mice. MT, the MRL/lpr mice treated with sterile water; Pred, the MRL/lpr mice treated with prednisone; FMT, the MRL/lpr mice treated with fecal microbiota transplantation.

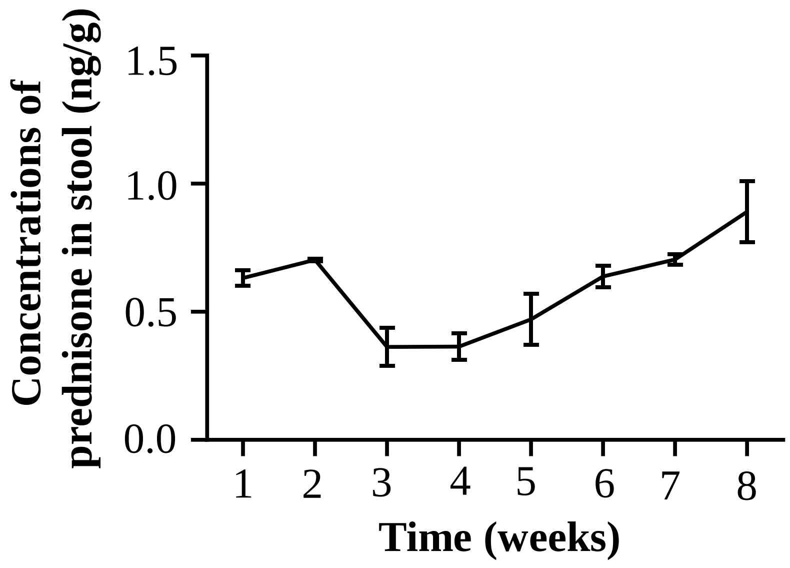

Figure S7 The concentration of prednisone in stool of MRL/lpr mice
